# The Patient and Carer Race Equality Framework: a model to reduce mental health inequity in England and Wales

**DOI:** 10.3389/fpsyt.2023.1053502

**Published:** 2023-05-05

**Authors:** Shubulade Mary Smith, Amna Kheri, Kevin Ariyo, Steve Gilbert, Anthony Salla, Tony Lingiah, Clare Taylor, Dawn Edge

**Affiliations:** ^1^Department of Forensic and Neurodevelopmental Science, Institute of Psychiatry, Psychology and Neuroscience, Kings College London, London, United Kingdom; ^2^South London and Maudsley NHS Foundation Trust, London, United Kingdom; ^3^UCL Medical School, University College London, London, United Kingdom; ^4^Institute of Psychiatry, Psychology and Neuroscience, King’s College London, London, United Kingdom; ^5^Steve Gilbert Consulting, Birmingham, United Kingdom; ^6^Oxytocin Learning Community Interest Company, Oxfordshire, United Kingdom; ^7^Kingston Hospital, Kingston upon Thames, United Kingdom; ^8^National Collaborating Centre for Mental Health, Royal College of Psychiatrists, London, United Kingdom; ^9^Division of Psychology and Mental Health Sciences, Faculty of Biology, Medicine and Health, The University of Manchester, Manchester, United Kingdom; ^10^Greater Manchester Mental Health NHS Trust, Manchester, United Kingdom

**Keywords:** competency framework, public mental health, mental health inequality, ethnic minority, epistemic injustice

## Abstract

The Patient and Carer Race Equality Framework (PCREF) is an Organisational Competence Framework (OCF), recommended by the Independent Review of the Mental Health Act as a means to improve mental health access, experience and outcomes for people from ethnic minority backgrounds, particularly Black people. This is a practical framework that should be co-produced with and tailored to the needs of service users, based on quality improvement and place-based approaches. We aim to use the PCREF to address the longstanding epistemic justices experienced by people with mental health problems, particularly those from minoritised ethnic groups. We will outline the work that led to the proposal, the research on racial inequalities in mental health in the UK, and how the PCREF will build on previous interventions to address these. By taking these into account, the PCREF should support a high minimum standard of mental health care for all.

## Background

People from ethnic minority backgrounds, particularly those who have Black African or African-Caribbean heritage, are disproportionately subjected to involuntary admission; have longer average lengths of stay in hospital; have higher rates of repeat admissions; may have higher rates of seclusion (1); are up to ten times more likely to be placed on Community Treatment Orders (CTOs) (2); are less likely to be offered psychological therapies and have higher drop-out from psychological therapy when they are offered it (3–5). These inequalities have developed in the context of White Eurocentric models of care, treatment and illness, as well as the historical context of colonialism.

In June 2017, the then British prime minister, Theresa May, commissioned the Independent Review of the Mental Health Act, chaired by Professor Sir Simon Wessely. This arose following longstanding concerns over the experiences of patients who had been detained under the Mental Health Act 1983 (amended 2007) (MHA), in particular those of Black African or African-Caribbean heritage. The more recent increases in MHA detention rates, between 2008 and 2016, added to concerns that service users from some ethnic minority backgrounds were having poorer experiences of care within mental health services.

Shortly before this announcement, in 2016, the Crisp Commission noted that patients and carers should be supported to play an even greater role in their own care as well as in the design, provision, monitoring and governance of mental health services (6). It was argued that just as there is a Workforce Race Equality Standard (WRES) to reduce inequalities in NHS staffing (7), there should also be a focus on the patient and carer perspective regarding care. To address this, the final report recommended that a Patients’ and Carers’ Race Equality Standard (PCRES), akin to the Workforce Race Equality Standard (WRES), should be piloted in mental health services/care to improve the monitoring and experience of care for people from minority ethnic communities. The Crisp Commission began a shift from focusing on workforce issues to focusing on patient and carer needs. What they did not suggest, however, was a mechanism for how this might be achieved. The Independent Mental Health Act Review suggested that an organisational competence framework might be the way to achieve this, the Patient and Carer Race Equality Framework (PCREF).

This paper explores how the PCREF could be a mechanism to work toward achieving epistemic justice, by creating the conditions for the collective knowledge base of racialised or ethnically minoritised people to be advanced. Indeed, the independent review of the MHA described the PCREF as ‘a new community-driven Organisational Competence Framework Tool’ which should enable Mental Health Providers (MHPs) to understand what practical steps they need to take to meet the needs of diverse ethnic backgrounds’. In this sense, the PCREF aims to function as a race equity and accountability framework, to support MHPs to demonstrate how they are meeting core legislative requirements relating to inequalities, and how they can improve the cultural competence of their organisation.

Key roles of the PCREF include supporting MHPs to improve their interaction with racialised or ethnically minoritised people and to ensure institutional accountability so as to improve experiences of care and treatment. One of the implicit challenges in this is acknowledging and overcoming the existence of power asymmetries, encompassing not only uneven power relations between service users/survivors and mental health professionals more generally, but also the compounded disadvantage as a result of systemic racism. Fricker’s theory of epistemic injustice provides a helpful foundation to explore and shape the forementioned roles of the PCREF, particularly the interactions and approaches to accountability between MHPs and people whose voices have been marginalised (8).

## Introduction

This paper sets out by reviewing some of the main approaches to reducing racial and ethnic inequalities in mental health care, including training, stepped care and culturally adapted provision. It does so with the aim of identifying the strengths of such approaches, while also making the case for alternative approach to removing racial disparities. The authors present the PCREF as an approach which can play a vital role in improving access, experience and outcomes for racialised or ethnically minoritised people by providing an accountability framework which is located at strategic levels for each MHP, and is connected to actions across an organisation, providing a form of accountability to patients and carers. This paper describes the process from which the PCREF materialised, and details broadly its component parts. Emphasis is placed on elucidating the priorities and principles which underpin the PCREF.

Epistemic injustice recognises that the creation of knowledge is never neutral (9). Fricker advanced ideas of epistemic injustice to elucidate the means by which marginalised groups are silenced, their opinions and experiences invalidated, “*specifically in her capacity as a knower*” (2007, p. 18), and how opportunities for alternative knowledge production and meaning-making are excluded. In very few places, if any, will this be more visible than in mental health services. Understanding whether and how the PCREF can help to address this imbalance for racialised or ethnically minoritised people is an additional purpose of this paper (10). The following section locates elements of the PCREF within the conceptual framing of epistemic injustice. It aims to illustrate how far the PCREF can go to addressing epistemic injustice in mental health, including the potential limitations and opportunities. A discussion of the main themes is presented, reiterating the need to prioritise race equality in mental health.

## Models aiming to reduce inequality in mental healthcare

There are existing interventions which attempt to reduce racial inequalities at both individual and organisational levels. For example, staff diversity training programmes have been implemented across various NHS trusts. These are intended to increase awareness of unconscious biases and to teach staff to mitigate their impacts on staff-patient interactions. However, existing evidence suggests that diversity (11, 12) and unconscious bias training in their current forms have limited effectiveness. Studies on unconscious bias training have found minimal positive (13) or even unintended, negative outcomes (14, 15), that may potentially reinforce bias. It is equally concerning that these approaches tend to focus on change at an individual level and have rarely been evaluated in terms of their effectiveness to produce change at the systemic level.

Given the absence of a strong evidence base, we must continue to explore and evaluate the underlying logic behind current and future interventions in the UK. Social epidemiologist Zinzi Bailey and her colleagues argue for an underlying approach based on “structural competency, cultural humility and cultural safety” (16). They cite policies employed within health and professionals’ training programs in several countries, such as Canada and New Zealand, as examples. Pre-registration training should encourage a “lifelong commitment to self-reflection and mutual exchange in engaging power imbalances along the lines of cultural differences.” Similarly, Hardeman and colleagues argue that health professionals already practising in the field can still “learn, understand, and accept” the current and historical basis of structural racism, encouraging cultural humility and furthering equity in clinical care and health research (17). The views above reflect the considerable ambitions behind these interventions and imply that an active learning process is needed to achieve them. While these programmes appear positive, it is unclear how such models would take effect in multicultural Britain, which developed from a unique and complex set of circumstances (18). Further, there remains very limited evidence of these training programmes being effective at reducing racial inequality at a system wide level (19).

Critics have questioned whether it is possible to facilitate comprehensive and sustainable learning within short-term interventions. As Byrne and Tanesini stated, one-off workshops and superficial cultural competence courses in medical education are insufficient to address unconscious racial bias (20). While common interventions such as mindfulness and implicit association tests (IAT) may be useful supplementary tools (21, 22), the potential for meaningful benefit can depend largely on the individual’s own motivations (23).

Other areas of research have focused on holistic interventions which offer a wider range of options to patients, reducing the effects of structural factors. Bhui and colleagues’ 2015 systematic review found that methods which improved access, engagement and outcomes for minority ethnic patients included complex interventions that engaged with social systems, stepped care and information and training for patients to support them to negotiate the mental health system (24). A further finding of this study was the importance of individual staff responsibility and monitoring which chimes with wider findings about the importance of individual and organisational accountability to achieving improved outcomes (25, 26). These findings were corroborated by the National Institute for Mental Health in England (NIMHE) community engagement project which used similar patient-focused strategies (27). Key improvements included better awareness and understanding of mental illness overall and how it is experienced by people from minoritised ethnic backgrounds; stronger engagement from services toward the community; better data provision and information dissemination to the community, and closer communication between the community and commissioners. Finally, user-led research has also emphasised that recovery is a dynamic process, requiring a flexible approach that focuses on engagement over a particular model of treatment (28, 29).

In terms of tangible policies, Penner and colleagues have suggested that a healthcare system of “aggregated” information could reveal patterns of neglect in individual patient care, such as one patient having repeated admissions for the same underlying issue (30). They argue that, by using algorithms tied to demographics such as socioeconomic status and race, it may be possible to identify mechanisms linked to systemic discrimination. This sounds appealing, however, the structure would need to include and be flexible toward patient reports (31). Of course, there is also the danger of this algorithm itself incorporating and reproducing unconscious biases (32).

Another in-depth, co-produced approach is the recently developed Culturally Adapted Family Intervention (CaFI) (33). The authors actively involved African-Caribbean people in their local communities and used Community-partnered participatory research (CPPR) (34) to find solutions related to psychosis and schizophrenia “with” the patients rather than “for” or “about” them (35). Using pre-existing models of family therapy, they added two further elements to strengthen its capacity to meet the specific needs of African-Caribbean service users and their families (36). These placed racism and discrimination, as well as alternative (non-Western) conceptualisations of mental illness, as central to the therapeutic process. The authors described the result as: “a focus on positive health and maintaining ‘gains’, better tailored relapse planning and using preventative strategies in an ‘assets-based’ approach toward community health. This catchment-specific approach enables setting bespoke criteria for good mental health outcomes in addition to standard ones.” As a result, CaFI complements several existing models (such as Open Dialogue; (37)) that seek to reduce power imbalances, but it encourages a more detailed consideration of culture, marginalisation and discrimination.

Overall, there is considerable scope to improve outcomes following targeted interventions to reduce mental health inequalities. As outlined above, interventions should focus on behaviours and foster curiosity and lifelong learning, which requires systemic support.

## A patient-focused race equality framework – the PCREF

The Mental Health Act African and Caribbean (MHARAC) working group of the Independent MHA Review asserted that, without a method for delivering them, the recommendations from the Crisp Commission would fail to be enacted, as with many other recommendations around race equality. The MHARAC group suggested that a competency framework approach was needed, similar to that taken by Roth and Pilling, a framework implemented to support the delivery of psychological services (38). Their paper sets out a method to summarise the evidence base, co-produce a set of competencies and develop these into a comprehensive model and training scheme. The idea arose for an organisational competency framework (the Patient and Carer Race Equality Framework, PCREF), practical guidelines, co-produced with and tailored to the needs of service users, based on quality improvement and place-based approaches.

The MHARAC group used evidence from a number of quantitative and qualitative sources to develop recommendations aimed at addressing the disproportionate detention of Black, Asian and Minoritised Ethnic people, particularly Black people, under the Mental Health Act 1983 (MHA). A mixed-methods approach helped to support this decision-making process:

1. Two Roundtable discussions at No.10 Downing Street with professional experts and Experts by Experience convened to explore the possible reasons for increased detention rates.
2. A series of 8 focus groups with people from Black, Asian and Minoritised Ethnic backgrounds were held across England and Wales.
3. A survey was conducted which received almost 2000 respondents from service users and carers (39–41).
4. Two working groups were set up to explore experiences with the MHA; one focused on people from Black African and Caribbean heritage (MHARAC) and the other focused on people from Asian and other Minoritised Ethnic groups (AME). A Service User and Carer Group (SUCG), oversaw and helped to integrate these workstreams. Some members of the SUCG were also core members on specific working groups.
5. As part of a suite of evidence reviews conducted for the overall Independent Review, the MHARAC group commissioned reviews on ethnicity and detention; substance use and workforce.
6. The MHARAC group used a formal consensus approach to derive coherent and evidence-based recommendations. This utilised a quasi ‘Nominal Group Technique’ which has been previously used to develop clinical guidelines (42). The process began with each person independently generating ideas and then sharing these through a series of discussions. The group synthesised these ideas, taking into account the quantitative research and qualitative evidence from the survey and focus groups, in addition to feedback from the working group members. The group then collectively formed recommendations and prioritised them, according to those most likely to produce real and lasting change in racial inequity in Mental Health Act detention. Numerous recommendations were generated, however, it was accepted that only a limited number of recommendations could be put forward for inclusion in the review.

Discussions centred on the following:-

1. There should be expectation of equality of access, experience and outcome. Co-production and the ability to learn about, understand and address the needs of those from a different culture to the practitioner should be fully embedded across mental health institutions, clinical and research, from service development to delivery and from hypothesis generation through to data collection, implementation and review.
2. Institutions should provide the necessary resources to collect high quality data that is sensitive to both diversity and intersectionality.
3. Commissioners and service providers should be supported to understand and address the needs of their local communities.
4. Closer attention should be paid to the inequalities that exist within some minoritized ethnic groups over the lifespan and their relationship with poorer social, clinical and economic outcomes, e.g., higher rates of adverse childhood experiences (ACEs) (43, 44).
5. Certain minoritized ethnic groups have 2-4x the average rate of school exclusions (45); although this likely stems from longstanding systemic inequality, evidence-based preventive interventions should be implemented for at risk pupils and their access to mental health support strengthened through non-coercive pathways.

One of the main considerations driving the thinking behind the development of the Patient and Carer Race Equality Framework (PCREF) were the findings from the Barnett et al. (46) meta-analysis. This not only replicated the findings of higher rates of detention in minority ethnic groups from previous studies (47, 48), but also noted substantial heterogeneity in the samples of Black and other ethnic minority groups entered into the studies, and that despite this, these groups were treated as homogeneous. Barnett et al. concluded that this crude approach to classification has prevented the translation of research involving minority groups into effective interventions to reduce inequalities in care.

Barnett et al. also reported the explanations given by the researchers when they observed higher rates of detention amongst ethnic minority groups. Notably, 47% of the reviewed articles provided either no evidence or very weak evidence to support their conclusions. This is a significant problem: these explanations have informed future research and interventions, yet the authors were unable to verify the strength of most claims. Many of the untested hypotheses involved stereotypical cultural and demographic assumptions of minoritised ethnic groups, including drug use, language barriers, illness expressed as violence and stigma. Barnett et al. concluded that this situation was “problematic,” having argued that such hypotheses possess little value when applied to heterogenous groups. Nevertheless, perspectives arising from these explanations are easily perpetuated and generalised into policy and commissioning.

The Barnett review indicated that for many years, researchers, clinicians and policymakers had been basing their decisions on flawed conclusions. It was postulated that this may explain why 40–45 years after the increased rate of detention of ethnic minority individuals in the UK was first noted (49–51), there have been few interventions which have reduced these disparities. The ensuing discussions formed the basis of the MHARAC group’s recommendations, which are outlined in brief below.

1. The rise in use of the Mental Health Act 1983 (amended 2007) has been influenced by factors at various levels of governance (52). To improve access, experience and outcomes for people from minoritised ethnic backgrounds, there should be better standards of feedback, review and quality improvement procedures in mental health services across the UK.
2. Rather than simply being a set of competencies decided centrally to address racial disparity to which an organisation must adhere, the PCREF is a model of working which involves an organisation learning how to develop a system, using a competency framework approach, to address the racial inequality in its services, based on the needs of the local population. An organisational competency framework defines the skills, knowledge, and characteristics required from staff in an organisation in order to fulfil the strategic priorities of that organisation, in this case achieving equality of access, experience and outcome in mental healthcare for people from minoritised ethnic groups. Organisational competency frameworks are commonly used to improve individual and organisational performance in large institutions, but have not been applied to racial equality in mental health. Applying this successful approach to improving equality within mental health organisations should reduce racial disparity in access, experience and outcomes.
3. The first step to achieving this will be to ensure a minimum standard of data collection to reduce variability in data collection (2). Following this, each mental health Trust must develop its own competencies (38), co-produced with the local community and tailored to meet the needs of its unique population. This requires a change in the usual approach to service development which traditionally involves developing a service, then inviting those who will use the service to comment on it (a consultation approach). With the PCREF, the expectation is that from the outset, the local population are invited to develop a collaborative partnership with the provider mental health organisation and together devise services that will better meet the needs of the local population. By being specifically designed with and tailored to the needs of the local population, mental health services are likely to be more accessible; the experience more acceptable and the outcomes better than the current service offer. By developing services in a more collaborative way, this approach gives those experiencing mental illness greater power in the relationship than currently exists between those providing mental healthcare and those receiving mental healthcare. The competency framework should include competencies focused on the policies, procedures and processes of the institution as well as the competencies expected of the individuals working within the organisation. Each organisation will go on to set co-produced internal standards based on these competencies and local data, with iterative review and an expectation of yearly improvement. It is this yearly improvement that can be subject to external scrutiny and quality assessment by regulatory and commissioning bodies.
4. In practise, there will be many commonalities between the competence frameworks developed by different organisations, however the priorities will differ depending on the needs of the local population. This has similarities to models proposed in the USA whereby benchmarking frameworks can be adapted and contextualised to local differences, but with a focus on cultural competency (53). Although developed to address issues within mental healthcare, this approach could be used in other organisations to support them in addressing racial inequalities.

The PCREF method is an organisational transformation approach using techniques based on/not dissimilar to those used to support transformation in large non-health organisations (54). Transformation is not simply organisational change, i.e., doing what the organisation does, but better, it is re-defining what the organisation does compared with what it does now (55). Focusing on achieving equity in mental health services, means that services will aim to move from providing good care to a proportion of its local population to providing excellent care to all of its population.

**Figure 1:**
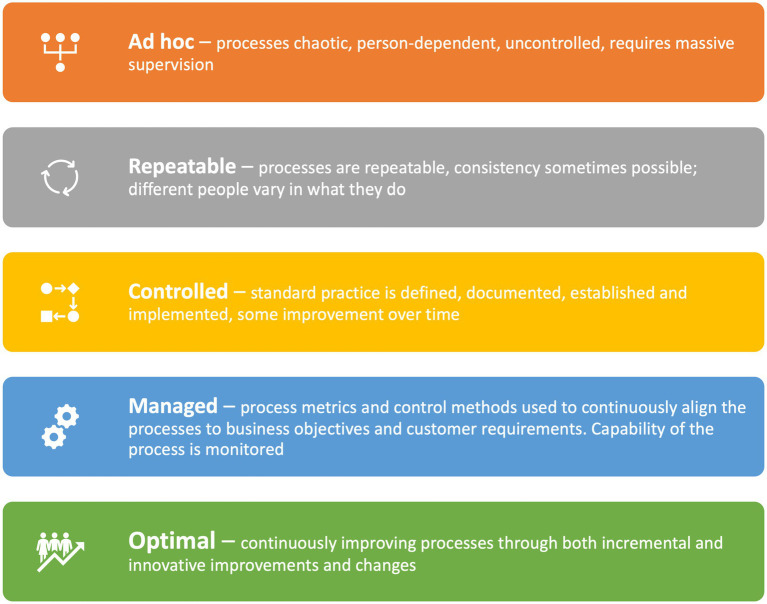
The characteristics of well-functioning organisations, from a maturity model perspective (56).

The PCREF aims for a maturity model approach (56) which can support an organisation with self-improvement, the focus of the improvement being the development of an equitable service. “*Maturity models (MM) are based on the premise that people, organisations, functional areas and processes evolve through a process of development or growth towards a more advanced maturity, going through a distinct number of levels….The basic concept underlying maturity is that mature organizations do things systematically, while immature organizations achieve their outcomes because of the heroic efforts of individuals using approaches that they create and use spontaneously*.” Maturity models are particularly useful for qualitative data where concrete and static solutions or circumstances are not available. A well-functioning organisation has in-built processes and procedures which are continuously improving; a poorly functioning organization has *ad hoc* processes and procedures which are uncontrolled; very person-dependent and easily corrupted. The aim of the PCREF is for organisations to become equity-led, that is, continuously improving processes designed to ensure racial equality in access, treatment and outcomes, through both incremental and innovative improvements and changes (Figure 1).

## Epistemic injustice and the PCREF

Epistemic justice encompasses ways of working and thinking which offer priority and value to people less powerful, including the autonomy for marginalised voices to be central in the development of conceptual resources about lived experience. By following an approach rooted in epistemic justice it is recognised that knowledge is socially formed, and regardless of an individual’s status or disposition, their testimonies should be validated and heard.

As a discipline which has favoured professional or clinical knowledge, mental health care is a key site for the study of epistemic injustice (57, 58). Such injustices can include being misrepresented, exluded from discussions or being silenced. Often the positions of psychologists, nurses, psychiatrists and other mental health professionals are viewed as reliable, whereas the positions of those with lived experience more often discredited. Framed within a system of Eurocentric frameworks and systemic racism, the silencing of racially minoritised service users is especially profound. It is against this background of micro-interactions and macro level dynamics that it is important to ask, what might the PCREF offer? Does it have a place in challenging epistemic injustice? If so, what might this be, and what could it look like?

As Russo points out, the focus in the application of Fricker’s work across mental healthcare has largely placed emphasis on the need ‘to listen better and empathise more’. Certainly, these are essential components which can allow racialised service users to indicate their experiences of care, mistreatment or trauma (59). However, across some race equality initiatives, such as those described earlier, (e.g., unconscious bias training), it is difficult to see a space for epistemic justice. It is therefore important to consider the sites where the PCREF can be more influential in fostering epistemic justice for racialised people and where there are likely to be limitations.

For the PCREF to become operational it will to some extent depend on data. This opens up a wider discussion about the way performance measures are identified locally and the kinds of data used as indicators. As Okoroji and colleagues have commented, there is a penchant within healthcare to rely on “positivist notions of a research ‘gold standard’ hierarchy, which marginalises experiential knowledge” (60). Numerical data on service access or outcomes in terms of equality monitoring can also be situated within positivist framework. Therefore, how much specificity and scope will the PCREF have to encourage MHPs to fully engage the testimonies of racialised lived experience, and the space and capacity for service user groups to be leading their own process of creating meaning.

Co-production is now viewed as one of the main approaches to engaging service user perspectives. Alongside partnership working, it is one of the features of the PCREF and the mechanisms through which engagement with racialised groups will occur. Ensuring at a strategic level that racialised people need to be involved in such processes is a step in the right direction. However, it is important to also recognise the possible limitations of co-production.

Rose and Kalathil refer to co-production as a ‘third-space’ which risks producing and reproducing already racialised hierarchies of knowledge (61). They present compelling cases of co-production which have perpetuated and engendered feelings of the ‘racialised mad’, ‘minoristised’ and ‘othered’. They conclude that genuine knowledge production cannot happen in places where the markers of dialogue are constrained, be this government or academic spaces, where strong traces of hierarchical, White, Eurocentric thinking remain. They guard against co-production being merely tokenistic and not truly informative. Rose and Kalathil suggest co-production will not be able to democrastise knowledge and efforts must be found in service user movements (61).

The idea that service users can be involved in the co-design of culturally adapted services has paralells with the CaFI project described earlier, and is presented in the PCREF as an area of organisational competency. Such an approach clearly has advantages; evidence indicates there can be improvements to experiences, especially when support has been found to be culturally affirming (62). At the same time, as with CaFI, involving service users in the design of services at the outset is an improvement on typical models found in healthcare. While the limitations to co-production have already been addressed, Russo eloquently describes how conventional applications of epistemic injustice ‘leave the concepts of ‘mental illness’ or ‘psychiatric disorder’ unquestioned’. This is a limitation which must be acknowledged with culturally adapted models which retain traditional biopsychosocial framing, and a point of consideration in relation to how far the PCREF can go to encouraging interventions which fully embrace the challenge of epistemic injustice.

The PCREF offers an opportunity to shape the way MHPs are accountable to patients and carers. However, a key emerging question relates to how far the PCREF can go in specifying the types of activities and data which will demonstrate some level of accountability. To some extent, this is not only a potential limitation of the PCREF but of the working parts which it has to incorporate within its framework, such as co-production. To address epistemic injustice, interventions must at least be considered across the remit of addressing testimonial and hermeneutical injustices.

The focus on partnership within the PCREF is a key area where epistemic justice could be realised. For example, establishing non-hierarchical partnerships with the Black Voluntary Sector (BVS), and service users movements, provides an opportunity for the collective knowledge base of Black people to be advanced. The BVS (also referred to as ethnocentric or ethno-specific support) has been identified as being particularly suited to racialised minority people. Keating has posited that the BVS offers the most relevant support to racialised groups because their work is based on different conceptual ideas about what it means to experience mental ill-health than do state services (63). Further, he argues that the BVS embraces the whole person when combating mental health problems rather than other models that define mental ill-health within a traditional biopsychosocial framework. Fernando makes a similar point when he suggests the BVS will bring in social and political issues within sessions, which are less likely to be brought into discussion within statutory provision. He suggests this means that a greater variety of personal problems can be considered including those which are racialised (64).

When considering Fricker’s distinction between testimonial and hermeneutical injustice, the BVS can be viewed as an alternative space of knowledge production. BVS organisations offer a place for service users to search for understanding and organically develop their own meaning frameworks and resources. Hermeneutical injustices can hinder testimonial justice, as those racially marginalised may not have the professional language, legislative knowledge or medicalised frames of references or access to resources to be utilised when in hierarchial epistemic encounters. In a similar way as culturally appropriate group advocacy (65) may offer solidarity to generating collective hermeneutic resources, which can positively impact testimonial justice, by providing a means for equitable participation and shared meaning making, fully embracing the opportunities for partnership working with the BVS could do the same. For example at the South London and Maudsley Hospital Foundation Trust, a pilot site for PCREF, service users and carers are trained in quality improvement techniques, in the same way as staff members, so that they can generate QI ideas that enable them to develop services (personal communication).

## Discussion

The consistent overrepresentation of Black African and Caribbean people in detention reflects wider systemic failures to respond to the needs of minoritised communities, and these disparities have not been reduced by major policy initiatives such as the 2005 Delivering Race Equality programme (66). We argue that these policies have not sufficiently understood the wide-ranging and intersecting factors that lead to structural inequity, which have resulted in inadequate prevention of mental health problems.

Not only could the PCREF achieve improved patient outcomes in access, experience and outcomes, but it also has the potential for economic benefits. An analysis suggested that in recent years, detentions under the MHA have increased substantially beyond the expected range, with the surplus alone costing approximately £75 million per year (52). This has had a disproportionately negative impact on Black people, who have consistently made up around 10% of total admissions over this period, despite only making up 3% of the UK population (52). This substantial inequality is a logical target for investment in order to ensure the NHS is more efficient in the long-term. If detention rates were proportionate to the percentage of Black African and Caribbean people in the population (3%), the basic annual cost to the NHS of detaining Black people would be reduced by an estimated £130 million (41). The PCREF should provide a means to help identify the causes of these inequalities and incentivise measures to address them.

The available evidence indicates the need for a PCREF approach. There is also a legal duty to address mental health inequalities. For example, the Public Sector Equality Duty under the Equality Act (2010) involves having “*due regard*” to the need to remove or minimise disadvantages suffered by people due to their protected (demographic) characteristics (67). Furthermore, in the United Nations’ recent evaluation of the UK’s adherence to human rights, they expressed concern about ethnic disparities in restraint, segregation and seclusion across settings, all of which take place under the MHA and the EHRC published guidance to support minimising and non-discriminatory use of restraint (68). The lack of improvement in ethnic disparities despite these imperatives, including the Public Sector Equality Duty, indicates that simply making recommendations is insufficient to bring about change. The PCREF represents a formal implementation method designed to aid transformation.

At its core, the PCREF will aim to further embed true co-production with service users and carers, and joint working between health, social care and voluntary sector services. These are also key priorities across national mental health policy, including the recent expansion of community mental health services (69), which draws on stepped care principles. While on the one hand it is recognised that it is too often the case that knowledge of people from minoritised ethnic backgrounds has drawn on the views of mental health professionals alone (70), the extent to which co-production can be a panacea to epistemic injustice, rather than just a modest improvement, will require each organisation to develop specific PCREF competences around how to ensure true co-production.

There are other areas where epistemic injustice might occur and where the PCREF might be able to support, for example, specific issues relating to cultural racism, when for example, the patient’s view of the progression of their care may differ from that of the professionals providing that care (71). An aim of the PCREF is to amplify the patient and carer voice in treatment planning. The experiences of racialised people growing up and living in a society within which they are systematically disadvantaged are all too often not even considered and when they are, may be dismissed. There is a challenge to ensure that dialogues include experiences of racism and take account of the systematic failure to include racialised people in knowledge production. As the PCREF will provide a lead for the development of comprehensive monitoring structures, ensuring closer alignment between local patients’ preferences and the care services provide to them, the opportunity is available to improve relationships between patients and clinicians, and increase the likelihood that they will engage with services, which may improve patient outcomes (72–74).

Finally, there is a need for continued advancement toward a mental health service that adequately reflects and engages with diversity in the UK. The need for this was identified as early as 1957, when a prominent ethnopsychiatrist outlined the divergence in psychosocial frameworks between first-generation Nigerian students and British psychiatrists (75). Mental health services still lack a mechanism to define, measure and evaluate cultural appropriateness. The required improvements in patient, carer and staff outcomes, as called for by the Old Problems, New Solutions report (76), cannot be realised without a bespoke framework that can regulate these issues at multiple levels of governance. The authors propose the PCREF can be part of the solution, but this will require genuine non-hierarchial relationships being developed with patients and carers and organisations. For a more meangingful attempt at addressing epistemic injustice in mental health this will require willingness to rethink the hegemonic model of ‘mental illness’ and bringing the knowledge of marginalised groups more to the forefront.

The PCREF aims to ensure that patients, carers, and the wider community, can be partners with mental health services in the delivery of support, care and treatment. This is the foundation of a high quality of care for patients of all backgrounds. Racial disparities in access, experience and outcome are both a symptom and a cause of unsatisfactory partnership working. By prioritising these areas, the PCREF should lead to more efficient mental health services, by promoting early intervention in the community thereby reducing the number of patients who present to secondary care in crisis.

## Conclusion

This paper has demonstrated the need for a PCREF, illustrating the disparities present in the access, experiences and outcomes to healthcare and making the moral and legal arguments to solve these. This practical framework, co-produced with and tailored to local service users, is based on quality improvement and place-based approaches. The PCREF focuses on four basic principles outlined in the Independent MHA Review: using a rights-based approach; focusing on dignity and patient autonomy; maximising patient choice and the right to advocacy; and standardising the path of least restriction and justification of therapeutic benefits.

These proposals are “good to think with.” Williams and Cooper suggest that mental health services require renewed emphasis on creating an environment of access to high quality care for all, including the consolidation of primary care, as part of healthcare delivery, and diversifying the healthcare workforce to more closely reflect the demographic composition of the patient population (77). The authors rightly term this as “putting more health into the delivery of healthcare.” We agree that more qualified research is needed to identify tailored methods of raising awareness of implicit bias and unconscious discrimination in mental healthcare and providing organisations with strategies to minimise its occurrence (77). This is included as part of the MHARAC recommendations, since it is recognised and asserted that despite the need, there is a scarcity of high-quality research and data collection. In terms of addressing social determinants, health care providers should indeed be “proactively engaged in connecting patients” with supportive social services (77). We believe that the PCREF can achieve this whilst making “effective use of local community resources and strengthening our surrounding communities,” as well as ensuring that “both community residents and institutions receive needed knowledge and technical skills to maximise the potential impact of interventions” (*ibid*). The model of the PCREF is therefore one of co-production with service users rather than a top-down approach.

We must emphasise that these recommendations are not calling for immediate radical restructuring. Rather, we are proposing incremental changes which should have impactful and, crucially, long-term accumulative effects. This is rooted in values of safe, equitable practise, ensuring that a high standard of care for all patients is at the heart of mental health service provision. The PCREF recommendation has been endorsed by the UK government who have funded four pilot sites.

## Data availability statement

The original contributions presented in the study are included in the article/supplementary material, further inquiries can be directed to the corresponding author.

## Author contributions

SS: conceptualisation, visualisation, methodology, investigation, writing – original draft, and supervision. AK and KA: visualisation and writing – original draft. SG, AS, TL, and DE: conceptualisation, methodology, and writing – reviewing and editing. CT: writing – reviewing and editing. All authors contributed to the article and approved the submitted version.

## Funding

This research paper was supported by the National Institute for Health Research (NIHR) Health Service and Delivery Research (HS&DR) Programme (project number 12/5001/62) and HTA Project: 16/167/76 awarded to DE. The funder had no role in study design, data collection, data analysis, data interpretation or the writing of the report. This paper also represents independent research supported by the NIHR Maudsley Biomedical Research Centre at South London and Maudsley NHS Foundation Trust and King’s College London. The views expressed are those of the author(s) and not necessarily those of the NIHR or the Department of Health and Social Care.

## Conflict of interest

Six authors took part in the 2019 Independent Review of the Mental Health Act (1983) African and Caribbean Group (MHARAC): SG as co-chair, SS as vice-chair, and KA, AS, TL, and DE as contributors. DE and SS are also co-applicants on the National Institute for Health Research-funded Culturally Adapted Family Intervention (CaFI) study.

The remaining authors declare that the research was conducted in the absence of any commercial or financial relationships that could be construed as a potential conflict of interest.

The handling editor CK is currently organising a Research Topic with the author AS.

## Publisher’s note

All claims expressed in this article are solely those of the authors and do not necessarily represent those of their affiliated organizations, or those of the publisher, the editors and the reviewers. Any product that may be evaluated in this article, or claim that may be made by its manufacturer, is not guaranteed or endorsed by the publisher.
